# COVID-19 pneumonia and target sign

**DOI:** 10.31744/einstein_journal/2021AI6564

**Published:** 2021-12-03

**Authors:** Lucas de Pádua Gomes de Farias, Daniel Giunchetti Strabelli, Gustavo Borges da Silva Teles

**Affiliations:** 1 UnitedHealth Group São Paulo SP Brazil UnitedHealth Group, São Paulo, SP, Brazil.

**Figure 1:**
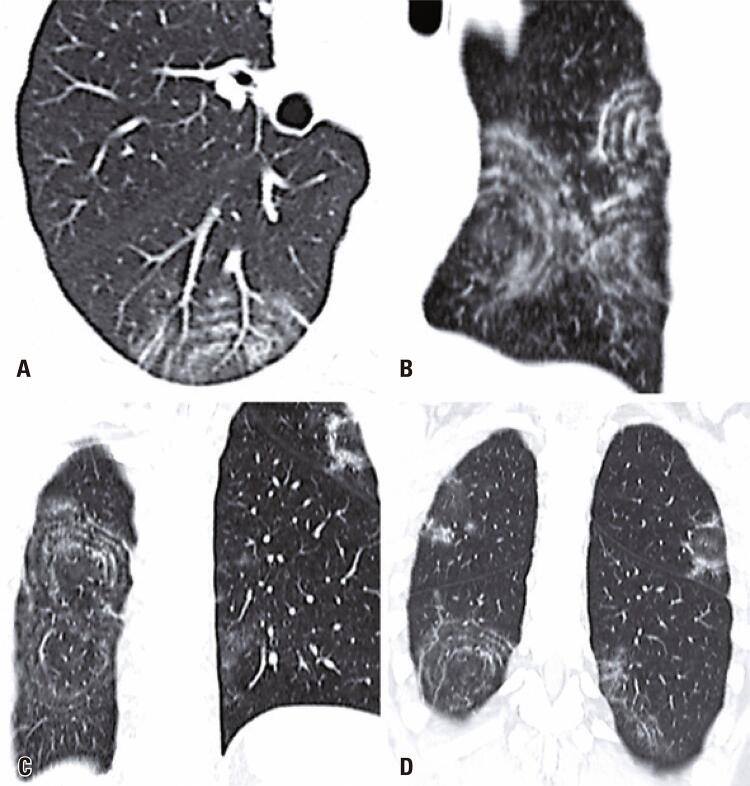
Chest computed tomography imaging (A) axial, (B) sagittal oblique, and (C and D) coronal oblique show the target sign characterized by multiple concentric ring-like opacities, with a central nodular peribronchovascular opacity, in a 49-year-old man with COVID-19 pneumonia. Note the reversed halo sign in the left upper lobe

A 49-year-old man came to our emergency department with a 2-day history of fever, cough, anosmia, ageusia and odynophagia. His past medical history included hypertension. At the time of this presentation, chest computed tomography revealed peripheral and bilateral ground-glass opacities, with some visible intralobular lines – typical findings of pneumonia caused by the severe acute respiratory syndrome coronavirus 2 (SARS-CoV-2). In addition, some findings revealed the target sign (Figure 1). The patient’s supportive treatment was continued, and reverse-transcriptase polymerase chain reaction (RT-PCR) confirmed the infection by SARS-CoV-2.

The target sign has been recently described and associated with organizing pneumonia and vascular features related to the viral infection.^(1-3)^ It is characterized by a nodular opacity in the center of a ring-like opacity, which can have ground-glass or consolidation attenuation, as well multiple concentric ring-like opacities.^(4)^ When only with one or two ring-like opacities, this sign is not specific to COVID-19, and the differential diagnosis includes conditions other than organizing pneumonia, such as metastasis and post-radiofrequency ablation zone.^(5,6)^ However, the manifestation with multiple concentric ring-like opacities (Figure 1) has only been reported so far in patients with COVID-19 pneumonia.^(4)^

We emphasize the importance of familiarizing radiologists with imaging findings of COVID-19 pneumonia to contribute to its diagnostic suspicion. The description of new radiological patterns, so far infrequent or little known, is of great value in the scenario of the SARS-CoV-2 pandemic, since the use of chest computed tomography has increased significantly in patients suspected to have COVID-19 pneumonia. It is noteworthy that further studies are still required to better assess the target sign and its accuracy in SARS-CoV-2 infection.
