# Supplementary material for: COVID-19 pneumonia and target sign
Source: Einstein (Sao Paulo). 2021 Dec 3;19:eAI6564. doi: 10.31744/einstein_journal/2021AI6564 (PMC8664281; doi:10.31744/einstein_journal/2021AI6564)
Supplement: Translation

# Translation: Pneumonia por COVID-19 e o sinal do alvo

**Authors:** Lucas de Pádua Gomes de Farias, Daniel Giunchetti Strabelli, Gustavo Borges da Silva Teles

**Affiliations:** 1 Brasil UnitedHealth Group, São Paulo, SP, Brasil.

Um homem de 49 anos procurou nosso pronto-socorro com história de 2 dias de febre, tosse, anosmia, ageusia e odinofagia. Sua história pregressa incluía hipertensão. No momento dessa apresentação, a tomografia computadorizada de tórax revelou opacidades em vidro fosco periféricas e bilaterais, com algumas linhas intralobulares visíveis, ou seja, achados típicos de pneumonia causada pelo coronavírus da síndrome respiratória aguda grave 2 (SARS-CoV-2). Além disso, alguns achados revelaram o sinal do alvo (Figura 1). O tratamento de suporte do paciente foi continuado, e a reação em cadeia da polimerase via transcriptase reversa (RT-PCR) confirmou a infecção por SARS-CoV-2.

**Figura 1:** Imagens de tomografia computadorizada de tórax (A) axial, (B) oblíqua sagital e (C e D) oblíqua coronal mostram o sinal do alvo caracterizado por múltiplas opacidades em forma de anel concêntrico, com opacidade peribroncovascular nodular central, em um homem de 49 anos com pneumonia por COVID-19. Observe o sinal do halo invertido no lobo superior esquerdo

O sinal do alvo foi recentemente descrito e associado à pneumonia em organização e a características vasculares relacionadas a infecções virais.^(1-3)^ É caracterizado por uma opacidade nodular no centro de uma opacidade em anel, que pode ser em vidro fosco ou em consolidação, e por múltiplas opacidades em anel concêntricas.^(4)^ Quando há apenas uma ou duas opacidades em anel, esse sinal não é específico da COVID-19, e o diagnóstico diferencial inclui outras doenças além da pneumonia em organização, como metástase e área pós-ablação por radiofrequência.^(5,6)^ Porém, a manifestação com múltiplas opacidades semelhantes a anéis concêntricos (Figura 1) foi relatada apenas em pacientes com pneumonia por COVID-19.^(4)^

Enfatizamos a importância da familiarização do radiologista com os achados de imagem da pneumonia por COVID-19, para contribuir com a suspeita diagnóstica. A descrição de novos padrões radiológicos, até então infrequentes ou pouco conhecidos, é de grande valia no cenário da pandemia de SARS-CoV-2, uma vez que o uso da tomografia computadorizada de tórax aumentou significativamente em pacientes com suspeita de pneumonia por COVID-19. Vale ressaltar que ainda são necessários estudos para melhor avaliar o sinal do alvo e sua acurácia na infecção por SARS-CoV-2.
